# Does Poor Sleep Quality Predict Functional Limitations?

**DOI:** 10.1093/geroni/igaa057.1378

**Published:** 2020-12-16

**Authors:** Elliot Friedman, Elizabeth Teas

**Affiliations:** Purdue University, West Lafayette, Indiana, United States

## Abstract

Previous work from our group (Friedman, 2016) linked sleep complaints to declines in mobility and risk of incident limitations over a 9-10 year follow-up among middle-aged and older adults. While these results suggest that poor sleep might undermine functional capacity, the self-report nature of the data leaves the robustness of this association unclear. The current study addressed this uncertainty by examining links between sleep and mobility limitations using subjective and objective assessments of both. Data were from the Midlife in the United States (MIDUS) study: the biomarker sub-sample (N = 664) from the original cohort (collected 2004-2006) and the Refresher cohort (collected 2011-2013). Sleep was assessed using the Pittsburgh Sleep Quality Index (PSQI; subjective) and 7 consecutive days of actigraphy (objective). Functional capacity was assessed by self-report of limitations and measured gait speed, grip strength, and chair stands. In linear regression models adjusting for demographic and health factors, lower PSQI scores (better sleep quality) predicted fewer reported limitations, stronger grip, quicker gait, and faster chair stands (all p<.01). Of the objective sleep metrics, time to fall asleep and time spent awake during the night predicted more self-report limitations, weaker grip (latency only), and slower gait speed and chair stands. These results extend our prior work by showing a) subjective sleep is linked to measured as well as self-reported physical function, and b) objective assessments of sleep predict reduced physical function, albeit to a lesser extent. They also brighten the spotlight on sleep as a key health determinant in older adults.

